# Carbon Nanotube Chemiresistor for Wireless pH Sensing

**DOI:** 10.1038/srep04468

**Published:** 2014-03-26

**Authors:** Pingping Gou, Nadine D. Kraut, Ian M. Feigel, Hao Bai, Gregory J. Morgan, Yanan Chen, Yifan Tang, Kara Bocan, Joshua Stachel, Lee Berger, Marlin Mickle, Ervin Sejdić, Alexander Star

**Affiliations:** 1 Department of Chemistry, University of Pittsburgh, Pittsburgh, PA 15260, United States; 2 Department of Electrical and Computer Engineering, University of Pittsburgh, Pittsburgh, PA 15261, United States; 3 Ortho-tag Inc., Pittsburgh, PA, United States

## Abstract

The ability to accurately measure real-time pH fluctuations *in-vivo* could be highly advantageous. Early detection and potential prevention of bacteria colonization of surgical implants can be accomplished by monitoring associated acidosis. However, conventional glass membrane or ion-selective field-effect transistor (ISFET) pH sensing technologies both require a reference electrode which may suffer from leakage of electrolytes and potential contamination. Herein, we describe a solid-state sensor based on oxidized single-walled carbon nanotubes (ox-SWNTs) functionalized with the conductive polymer poly(1-aminoanthracene) (PAA). This device had a Nernstian response over a wide pH range (2–12) and retained sensitivity over 120 days. The sensor was also attached to a passively-powered radio-frequency identification (RFID) tag which transmits pH data through simulated skin. This battery-less, reference electrode free, wirelessly transmitting sensor platform shows potential for biomedical applications as an implantable sensor, adjacent to surgical implants detecting for infection.

## Introduction

The pH of a solution can have a significant effect on chemical processes, therefore both the measurement and control of pH is important for materials, life, and environmental sciences. Many physiological processes can be affected and monitored through pH changes such as microbial infection^1^, tumor metastasis^2^, and wound healing^3^. For example, detecting pH changes on surfaces of implanted medical devices can reveal acidosis associated with bacteria colonization of the implant. Although non-invasive methods such as X-ray scintillation have been recently demonstrated^4^,^5^, pH monitoring *in vivo* using implanted sensors represents a significant technical challenge. Glass electrodes generally implemented for pH measurements require a reference electrode and suffer from leakage of electrolytes in addition to a number of other errors^6^. The field of ion-selective field-effect transistors (ISFETs) which started more than 40 years ago^7^ has promised development of rugged, small, rapid response pH sensor devices. Also, ISFETs would not require hydration and would be inert toward harsh environments. While there are numerous advantages of using ISFETs, one major limitation of the technology involves the requirement of a reference electrode ultimately limiting their size^7^. Continued efforts have been made to develop on-chip miniaturized reference electrodes^8^,^9^,^10^,^11^,^12^,^13^; however because of these electrodes, ISFETs suffer the same limitations as conventional glass pH electrodes. A sensor device that alleviates the need for a reference electrode, glass membrane, and an auxiliary power supply would be ideal for *in vivo* medical applications.

We demonstrate here that carbon nanomaterials could alleviate the aforementioned problems. Specifically, a chemiresistor based on carbon nanotubes (CNTs) only requires measurement of the resistance of the nanotube network as a function of ion concentration, ultimately eliminating the need for a reference electrode. The low-cost, rugged properties of CNT-based sensors, and the excellent electronic properties of single-walled carbon nanotubes (SWNTs) offer great potential to the development of stable, miniaturized, implantable pH sensors.

There are previous examples of CNTs used for pH sensing^14^,^15^,^16^. Although these devices are successful in sensing pH change there is relatively no selectivity between hydronium ions (H_3_O^+^) and metal cations. To achieve the required specificity to H_3_O^+^, oxidized single-walled carbon nanotubes (ox-SWNTs) were functionalized with a conducting polymer (CP). Recently, CPs have been studied for detection of pH^17^,^18^,^19^, as their electrical properties strongly depend on their degree of protonation, and interestingly have shown very fast response when the sensing process occurs on the surface of the polymer^20^. However, a major problem of all organic conductors is their limited chemical stability. CNTs can help to stabilize polymers, increasing the lifetime of the sensor device^21^, while additionally increasing the aspect ratio of the polymer for facile integration into micrometer sized electronics. Polypyrrole (PPy) and polyaniline (PANi) polymers have been combined with nanotubes toward the development of solid state pH sensor devices^22^,^23^,^24^, however these electrochemical based devices require the use of a reference electrode which limits many advantages of the system, specifically applications toward implantable biosensors.

The fabrication process can involve applying coatings necessary for the protection and longevity of implantable devices placed inside the body. Coatings like Nafion and hydrogels are often added to overcome foreign body responses which can disrupt the implanted device's function^25^,^26^,^27^. Nafion films require an annealing step which, depending on the polymer type, can involve exposing the devices to temperatures as high as 210°C^28^,^29^,^30^. These high temperatures can have adverse effects on the conductive polymer material chosen to make the device. PPy and PANi polymers are shown to degrade and loose conductivity at temperatures exceeding 70°C over time^31^,^32^,^33^,^34^,^35^. Therefore, we have chosen poly(1-amino anthracene) (PAA) for our conductive polymer element. PAA is reported to have high electroconductivity and thermostability^36^. PAA films have already been shown to have Nernstian response in the 1–12 pH range with no measured interference with alkaline counter ions^37^.

We have developed a pH sensitive device by combining ox-SWNTs with PAA that does not require the use of a reference electrode. The pH levels can be monitored electrically by measuring the conductance across the ox-SWNT/PAA network, which changes linearly with respect to the concentration of hydronium ions in solution. The low cost, extremely small size, and sensitivity of this device offers great promise toward the development of a commercially viable, implantable, solid-state pH sensor device. As proof of principle we have attached our pH sensor to an RFID tag prototype that is designed to be placed subcutaneously on or near surgical implants. These devices have shown the ability to be wirelessly powered and detect changes in pH through simulated human skin.

### Functionalization of ox-SWNTs by electropolymerization

**Figure 1:**
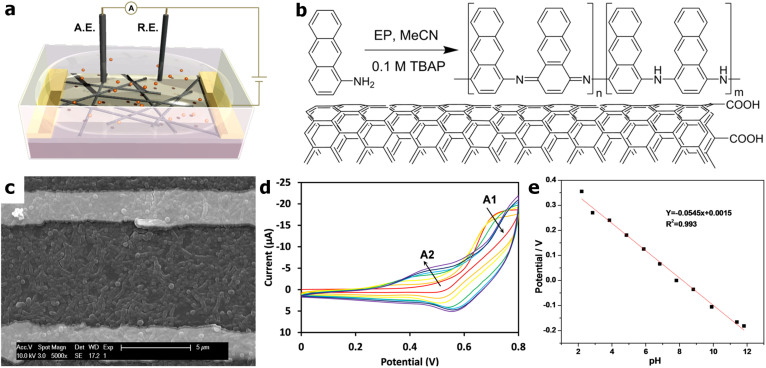
PAA/oxidized single-walled carbon nanotube (ox-SWNT) device fabrication and characterization. (a), Generalized schematic of electropolymerization (EP) of PAA onto ox-SWNTs using the nanotube network as the working electrode (W.E.) with a Ag/AgCl quasi reference electrode (R.E.) and a Pt wire auxiliary electrode (A.E.). (b), Synthesis of poly(1-aminoanthracene) (PAA) from 1-amino anthracene (AA) by electropolymerization on ox-SWNTs. (c), Scanning electron microscopy (SEM) image of the ox-SWNT/PAA network in-between two finger electrodes. (d), Cyclic voltammetry (CV) curves of EP of PAA onto ox-SWNT. As the number of cycles increases, from 1 (red) to 30 cycles (purple), the oxidation peak for the AA monomeric unit (A1) disappears and the peak for PAA (A2) appears. (e), Calibration curve derived from potentiometry measurement of ox-SWNT/PAA exposed to various pH solutions (vs. Ag/AgCl electrode).

SWNTs were used to create semiconducting networks between interdigitated gold electrodes on silicon chips. Once the ox-SWNT device was made, a conductive polymer, PAA, was electropolymerized on the surface of the SWNTs. PAA was synthesized using a previously published electropolymerization (EP) procedure^38^. Figure 1a depicts a typical device setup for EP of 1-amino anthracene (AA) to PAA. In this three electrode electrochemical cell, a network of ox-SWNTs is configured as the working electrode (W.E.). The potential of the W.E. is varied with respect to a quasi-reference electrode (R.E.) (Ag/AgCl), while the Pt wire auxiliary electrode (A.E.) is used to monitor the current produced without changing the potential of the R.E. By sweeping the potential of the W.E. while the system is submerged in an electrolyte solution (TBAP in MeCN) containing AA monomeric units, PAA is formed (Fig. 1b). An SEM image of the resulting PAA/ox-SWNT material is presented in Figure 1c. More specifically, by sweeping the potential from 0 to +0.8 V at a sweep rate of 0.05 V/s, one cyclic voltammetry (CV) cycle is completed, and thus the formation of PAA. The resulting current, plotted *versus* potential in Figure 1d, provides some information about the EP process. With an increase in the number of cycles from 1 (red) to 30 cycles (purple), peaks for the oxidation of the monomeric unit (A1) disappear and a peak for dimer and oligomer units (A2) appears. Potentiometry measurements yield a response to change in pH that approaches the Nernst limit for hydronium ion detection (Fig. 1e)^39^.

We found that the number of EP cycles has a significant effect on pH response, which was correlated with the thickness of the PAA coating as revealed by SEM analysis (Supplementary Fig. S1). The PAA coating thickness generated from 0–10 cycles does not adequately cover the ox-SWNT network and results in a pH responsive device with a sensitivity of 0.002 ± 0.001. Exceeding 60 EP cycles results in a PAA coating too thick and the individualized ox-SWNTs can no longer be visually distinguished and these devices cease to adequately respond to pH changes. There is a middle range in which the number of EP cycles generates a coating that is optimal. This optimal range of EP cycles is 11–60 EP cycles. The resulting PAA thickness in this range of cycles generates a coating that adequately covers the ox-SWNT network while allowing the individual SWNTs features to remain. The FET devices with 11–60 EP cycles results in device sensitivities of 0.14 ± 0.08. These results suggest that more PAA material will increase the pH response as long as the coating is not too thick to effectively transfer the effect of the pH solutions through to the surface of the ox-SWNTs. Energy dispersive X-ray spectroscopy (EDX), X-ray photoelectron spectroscopy (XPS), and atomic force microscopy were performed to analyze polymer composition and thickness (Supplementary Tables S1 and S2, Fig. S2–S4).

To further optimize the pH response the oxidation of the SWNTs was evaluated (Supplementary Table S3 and Figure S5). The SWNTs were oxidized by exposure to concentrated H_2_SO_4_/HNO_3_ (3:1) and sonication for 1–2.5 h. The length distribution was measured from TEM images and the carboxylic acid loading was determined using the Boehm's titration method^40^. The optimal carboxylic acid loading that remained electrically viable for the construction of FET devices was determined to be at 2 h of oxidation. The carboxylic acid loading continued to increase over 2 h but the electronic properties were no longer useful.

### Field-effect transistor and chemiresistor testing

**Figure 2:**
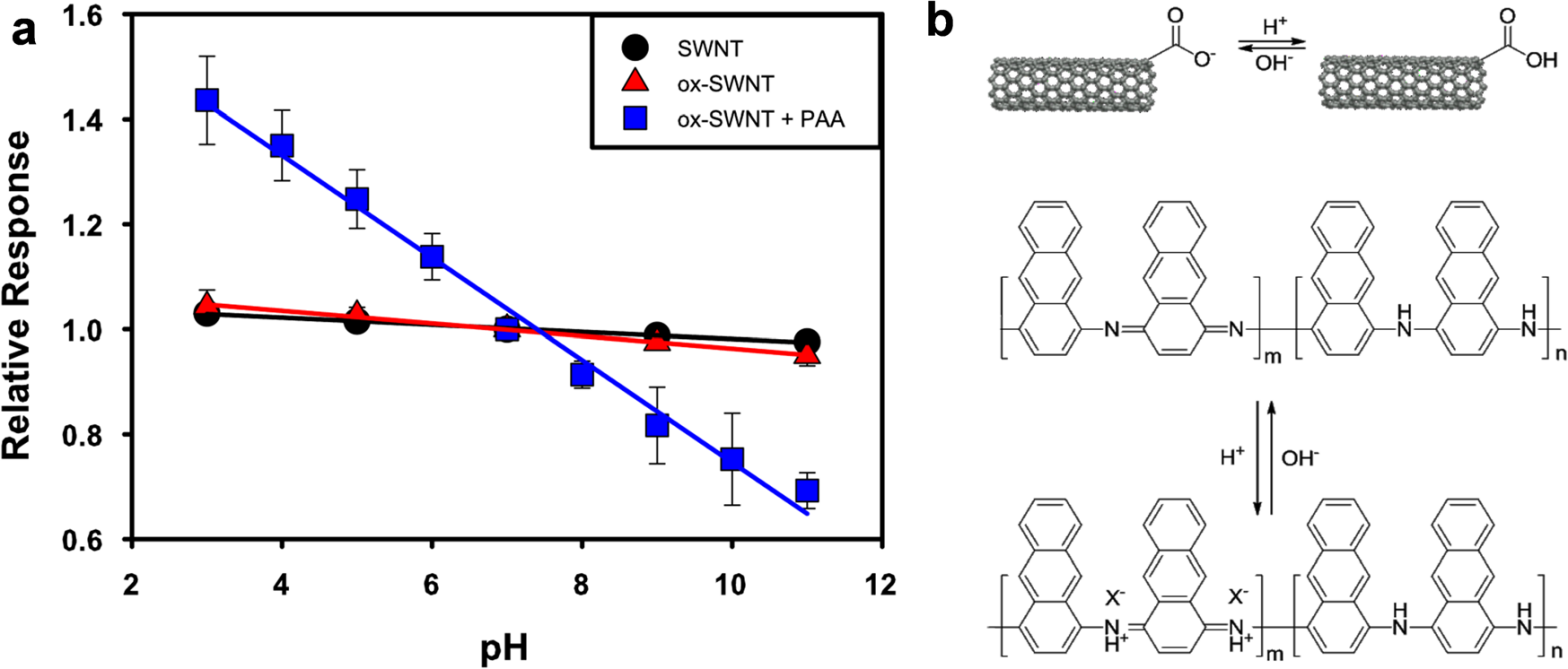
(a), Comparison of relative response for pristine (un-oxidized) SWNTs (SWNT, black), oxidized SWNTs (ox-SWNT, red) and ox-SWNT + PAA (ox-SWNT/PAA, blue) used as a conductance pH sensor. (b), Proposed schematic illustrations of pH sensing mechanism for ox-SWNT (top), PAA (bottom).

The conductivity of pristine SWNTs is affected by the presence of OH^−^ and H_3_O^+^ species in solution and will thus have a pH response. Although the conductivity of SWNTs will change upon exposure to different pH solutions, we wanted to create the most pH sensitive devices. Therefore, different types of SWNT-based devices were tested (Fig. 2). Figure 2a compares pH response of devices fabricated from SWNTs, ox-SWNT, and PAA coated ox-SWNTs (ox-SWNT/PAA). The presence of oxygen containing groups on the surface of SWNTs most likely plays a role in pH response. Therefore, the added carboxylic groups on the ox-SWNT will enhance the response. For increased pH response, the conductive polymer PAA was added. It is apparent that by combining the two types of materials synergetic effects are observed. The polymer contributes to the device selectivity, while the carbon nanotubes provide a sensitive and robust platform necessary to chemically stabilize the polymer. As shown in Figure 2b the combined response of the protonation of ox-SWNT carboxylic groups, the hydrogen bonding interaction between the CP and the ox-SWNT, and the charges present on the CP all contribute to an overall maximized pH responsive device.

**Figure 3:**
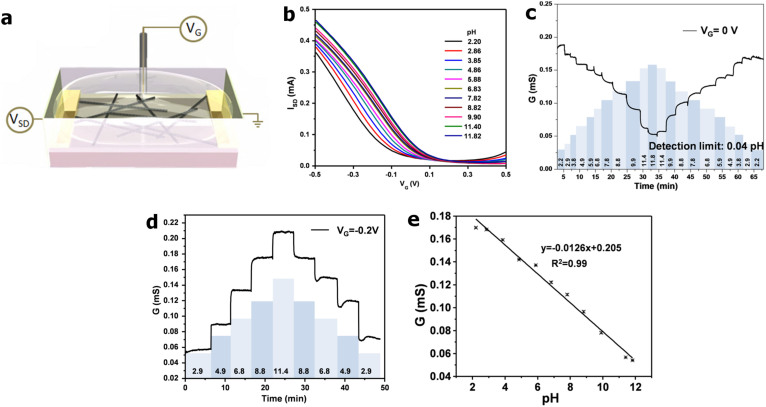
(a), Schematic illustration of setup used for field-effect transistor (FET) characteristics measurements. A constant source drain voltage is applied across the nanotube network while the gate voltage (V_G_) is swept between −0.5 and +0.5 V. (b), FET transfer characteristics (i.e., source drain current (I_SD_) vs. applied liquid gate voltage (V_G_)) of a device functionalized by ox-SWNT/PAA exposed to various pH solutions. (c), Conductance (G) vs. time measurements of ox-SWNT/PAA device exposed to various pH solutions under a floating gate condition (pH values shown in banded bars). (d), Conductance vs. time measurements of the same device as in panel **c** under constant −0.2 V gate voltage, showing a reversed G ~ pH trend compared to panel **c**. (e), Calibration of G vs. pH for pH response shown in panel **c**.

Upon optimization of the SWNT chemical oxidation (2 h) and the number of EP cycles (40), devices were created and tested for pH response (Fig. 3). FET measurements were performed in order to investigate the SWNT device response. These measurements were done by passing a constant source drain voltage through the nanotube network (V_SD_ = 50 mV) and measuring its current (I_SD_) while sweeping the voltage applied through a liquid gate (V_G_). These characteristics (I_SD_ vs. V_G_) provide information about the semiconductor properties of our devices.

Figure 3a is a schematic illustration of how FET characteristics were measured. The gate was applied in solution through a Ag/AgCl reference electrode (liquid gating). Figure 3b shows how these characteristics change when the device is exposed to various pH solutions ranging from 2 to 12. The FET curve shifts to more positive gate voltage as the pH of the buffer solution increases. This shift is indicative of there being more negative charges generated on the surface of the nanotubes due to deprotonation of carboxylic groups on the ox-SWNTs in addition to more electron rich species present in the PAA coating. This response is expected from a p-type semiconductor in which the charge carriers are holes. Therefore, if there is added negative charge from the PAA material, the amount of negative gate voltage needed to generate the same transfer characteristics will be less. The opposite is true for acidic conditions where the surface of the ox-SWNT will be exposed to positive charges which will require more negative gate voltages to generate the same magnitude of I_SD_. The resulting surface charges from exposure to pH solutions are acting as an additional gate in this scenario and the overall transfer characteristics stem from the charge induced by both the pH change and the applied V_G_. This trend was also observed in other studies using SWNTs to monitor pH changes^23^,^41^,^42^,^43^,^44^. Specifically, Lee et al. demonstrated that by coating ox-SWNTs with a PMMA dielectric layer an enhanced gating effect resulted, causing the conductance of the ox-SWNTs to increase with an increase in solution pH^43^. Back et al. presents a detailed study of ox-SWNT transfer curve dependence on pH^41^. The same FET transfer curve behavior is observed by their group as what is presented here. Through systematic control experiments Back et al. conclude that the change in the transfer characteristics arise from charges found at the surface of the ox-SWNTs thus inducing an offset in the gate voltage.

In keeping with the goal of creating a miniature, passively powered pH sensor, a chemiresistor architecture is preferable since the gate electrode is eliminated. The sensitivity of the chemiresistor to various analytes can be determined with relative ease. Conductivity measurements were acquired by holding the V_SD_ passing through the ox-SWNT/PAA network constant and measuring the resulting I_SD_ as a function of analyte. The pH response of the ox-SWNT/PAA device was tested over a large range of pH solutions under floating gate conditions (no gate voltage applied) (Figure 3c).

The resulting conductance response matched that of the positive gate voltage region seen in the FET curves in Figure 3b. As the solution pH increases the device conductance decreases. This is a very interesting result since one would assume that under floating gate conditions the gate voltage would be zero. To test this result further, a gate voltage of −0.2 V was applied to the same device (Fig. 3d). The resulting conductance response was in accordance with the FET curves in the negative gate voltage region (Fig. 3b). These experiments prove that when operating the ox-SWNT/PAA devices as a chemiresistor (no gate voltage) the response measured is that of the material with more n-type characteristics. This result also stresses the importance of investigating charge transfer characteristics in these semiconductor devices in order to more accurately identify the mechanism of analyte response.

Furthermore, the stability and reproducibility of the device was measured (Supplementary Fig. S6). The pH response of the same ox-SWNT/PAA device was tested initially then 120 days later and produced a calibration with the same sensitivity. The decrease in overall conductance for the same pH measurement indicates that a single point calibration would be required for accurate pH readings over many days. This drift, which is a common problem known in CNT-based sensors, was only observed after many days whereas conductance readings held steady for a device exposed to pH 5 and pH 3 for 2 and 1.5 h, respectively.

Results thus far have shown the sensitive response to a wide range of pH values but if these miniature devices are to be implemented for biosensing, the pH range of interest will be much narrower. This ox-SWNT/PAA device also shows sensitive response to physiologically relevant pH changes with response times as low as 3 s. Li et al. has reported a 11 s response time of ox-SWNT devices upon exposure in the pH 7 regime^14^.

Control experiments were performed in order to further evaluate sensor performance and elucidate the mechanism of detection. In order to determine that the funtionalization platform is specific to hydronium ions, Ca^2+^ and Na^+^ were tested as control analytes (Supplementary Fig. S6). Previous studies from our group showed bare SWNT sensor devices respond to metal cations resulting in an increase in conductance with increased metal concentration^45^. The ox-SWNT/PAA device did not respond to concentrations of such ions in the range of 10^−12^ to 10^−6^ M, comparable to the range of concentrations of H_3_O^+^ ions tested.

### Implantable, wireless pH sensor

**Figure 4:**
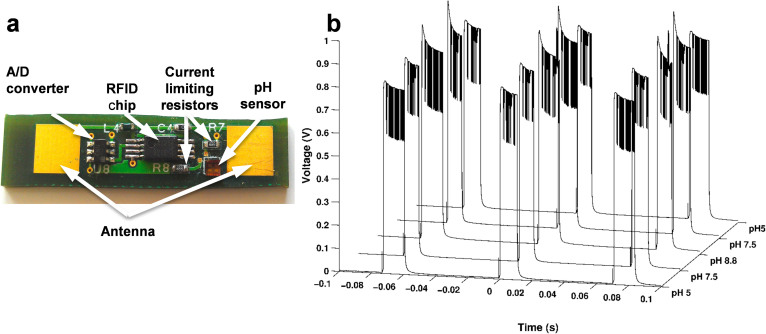
(a), Image of the passively powered, implantable RFID tag pH sensor prototype. (b), Voltage vs time data collected in varying pH solutions. Each trace consists of three 12 ms pulses.

The application of this device was illustrated by attaching it to a wirelessly powered, implantable RFID tag prototype (Fig. 4a). A custom touch probe was designed to passively power and receive data from the RFID tag. The touch probe connects to a commercial RFID reader and works, in contact, through a 1 cm tissue phantom to obtain data from the RFID tag. The RFID tag was also connected to an oscilloscope which recorded the readings from the tag directly during testing (Supplementary Fig. S7–S13). The voltage across the sensor was observed to increase with exposure to solutions of higher pH, as expected due to a decrease in sensor conductance with higher pH (Fig. 4b). These results demonstrate the ability to passively power the sensor and wirelessly obtain readings of changes in sensor conductance, suggesting the possibility of *in vivo* monitoring with a fully implantable, battery-less device.

## Conclusion

Through optimization of each material used in the fabrication of the ox-SWNT/PAA device, we were able to achieve a miniature, sensitive, stable, and reproducible solid-state pH sensor. Using this device as a chemiresistor, we can achieve sensitive pH response without the need for a reference electrode. Removing the reference electrode relieves the size limitation of this device. A smaller device can more easily be implanted at a location of interest inside the body or become incorporated with other implanted devices such as prosthetics. This was shown to be possible through the implantable RFID tag prototype. The added stability of carbon nanotubes can enable in-situ monitoring in other medical and environmental applications. For example, monitoring gastrointestinal reflux where the sensor can be exposed to extreme acidic conditions^46^,^47^. The incorporation of miniature CNT-based sensors onto remote controlled platforms could enable remote sensing of hard to access or hazardous environments while the low power requirement of these devices is conducive to wireless operation.

## Methods

### Materials

Pristine single-walled carbon nanotubes (SWNTs) were purchased from Carbon Solutions, Inc. (P2). 1-aminoantracene (AA), anhydrous acetonitrile (MeCN), and tetrabutylammonium perchlorate (TBAP) were obtained from Sigma Aldrich. Buffered pH solutions were prepared through Britton-Robinson methods^48^ from pH = 2 to pH = 12. The pH of the buffered solution was measured using a Mettler Toledo Seven Multi pH meter. The pH meter was calibrated with standard buffered solutions (pH 4, pH 7 and pH 10) obtained from J. T. Baker.

### Preparation of conductive ox-SWNTs

SWNTs (13 mg) were dispersed in 20 mL of concentrated H_2_SO_4_/HNO_3_ (3:1). The mixture was subsequently sonicated for 2 h at 40°C in an ultrasonic bath (5510 Brasonic) to yield ox-SWNTs with lengths around 400 nm. Carboxylic acid groups were confirmed through FTIR spectroscopy and using the Boehm's titration method^40^. Upon completion, the mixture was added dropwise to 200 mL of cold distilled water and then filtered through 0.2-μm pore size PTFE (Teflon) laminated filter paper and then washed with water until no residual acid was present.

### Sensor Device Fabrication

Silicon chips with 300 nm thermal oxide layer and pre-fabricated interdigitated Au electrodes (MEMS and Nanotechnology Exchange) were wire-bonded into a 40-pin CERDIP package, followed by passivation of the system with epoxy (EPO-TEK, Epoxy Technology, MD USA). For FET experiments, suspensions (30 μL) of ox-SWNTs in N,N-dimethylformamide (DMF) were deposited onto the Si chips via the dielectrophoresis (DEP) technique (Angilent 33250A waveform generator, with an applied ac frequency (300 kHz), bias voltage (10 Vpp), bias duration (30 s))^49^ and allowed to dry in ambient conditions. For continuous conductance measurements, suspensions (0.3 μL) of ox-SWNTs in DMF were drop-cast onto the Si chips. PAA was subsequently added to the ox-SWNT network *via* electropolymerization (EP) of the monomeric units, AA, into the polymer, PAA^38^.

EP was performed using a CH Instruments electrochemical analyzer with ox-SWNTs configured as the working electrode in a three-electrode single compartment electrochemical cell. A platinum wire and a Ag/AgCl quasi-reference electrode were used as the auxiliary and reference electrodes, respectively. Anhydrous acetonitrile was used as the electrolyte solution which contained the supporting electrolyte, TBAP (0.1 M), and the monomeric units, AA (1 and 10 mM). PAA was prepared on the ox-SWNT film using cyclic voltammetry by sweeping the electrode potential between 0 and + 0.8 V at rate of 0.05 V/s. The electrodes were conditioned in an aqueous solution containing a pH 5 buffer for 24 h prior to testing. The formation and morphology of PAA was monitored through scanning electron microscopy (SEM). SEM was performed on a Philips SL30 FEG microscope at an accelerating voltage of 10 keV. Through characterization, the optimal number of EP cycles was determined to be 40 cycles.

### Electrical Measurement

For pH-solution sensing, devices fabricated as explained above were modified with a custom vial adhered to the top of the CERDIP package containing the chip. The devices were exposed to 500 μL of varying buffered pH solutions. Conductance of the ox-SWNT/PAA network was measured versus time while holding a constant voltage (V_SD_) of 50 mV using a Keithley 2400 source meter. Field-effect transistor (FET) measurements were taken using two Keithley 2400 source meters. One to hold the constant bias voltage (V_SD_ = 50 mV) and the other to sweep the gate voltage (V_G_ = −0.75 to 0.75 V) through a Ag/AgCl reference electrode in buffered pH solutions. Potentiometry measurements were carried out using a CH Instruments electrochemical analyzer in a three-electrode setup similar to the EP process with the devices as the working electrode, a Ag/AgCl reference electrode and a Pt counter electrode.

### RFID Implantable Sensor

The sensor was mounted on a prototype implantable tag with circuitry to detect and communicate changes in the sensor conductance. The sensor was connected in a voltage divider to measure voltage changes related to changes in sensor conductance. The voltage across the sensor was read by an RFID chip (EM4325, EM Microelectronic) via an analog-to-digital converter. A custom touch probe connected to an RFID reader (IF2 Network Reader, Intermec) was used to wirelessly power the components on the tag and obtain readings of the sensor voltage from the RFID chip. The voltage across the sensor on the tag was also continuously measured using an oscilloscope. Wireless readings were performed through a tissue phantom designed to mimic the properties of human skin at the transmission frequency of 915 MHz. The sensor was exposed to solutions of pH 5, pH 7.5, and pH 8.8.

## Author Contributions

P.G., N.D.K, I.M.F., G.J.M., and A.S. wrote the main manuscript text. P.G., I.M.F., H.B., G.J.M., Y.C., Y.T. fabricated and characterized carbon nanotube-based pH sensors and prepared figures 1–3. K.B., J.S., L.B., M.M. and E.S. designed RFID implantable sensor, analyzed the sensor data and prepared figure 4. All authors reviewed the manuscript.

## Supplementary Material

Supplementary Information Supplementary Information File
