# Supplementary material for: Carbon Nanotube Chemiresistor for Wireless pH Sensing
Source: Sci Rep. 2014 Mar 26;4:4468. doi: 10.1038/srep04468 (PMC3966035; doi:10.1038/srep04468)
Supplement: Supplementary Information — File [file srep04468-s1.pdf]

Supplementary Information

Carbon Nanotube Chemiresistor for pH Sensing

*Pingping Gou,[1] Nadine D. Kraut,[1] Ian M. Feigel,[1] Hao Bai,[1] Gregory J. Morgan,[1] Yanan Chen,[1] Yifan Tang,[1] Kara Bocan,[2] Joshua Stachel,[2] Lee Berger,[3] Marlin Mickle,[2] Ervin Sejdić,[2] and Alexander Star[1,]**

[1]Department of Chemistry, University of Pittsburgh, Pittsburgh, PA 15260, United States

[2] Department of Electrical and Computer Engineering, University of Pittsburgh, Pittsburgh, PA 15260, United States

[3] Ortho-tag Inc., Pittsburgh, PA, United States

Email: astar@pitt.edu

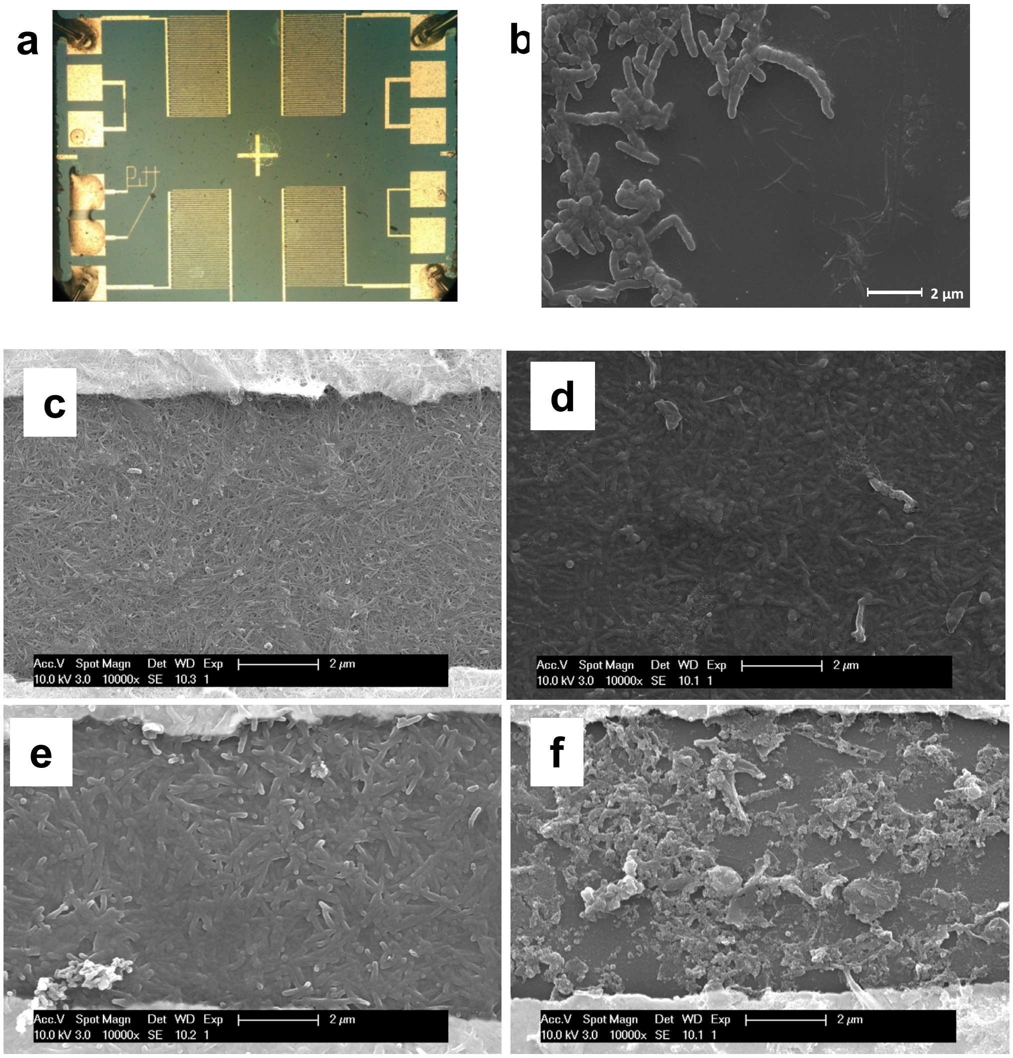

Figure S1. **a,** Optical microscopy image of the Si/SiO_2 sensor chip (2 mm x 2 mm) with four interdigitated gold electrode devices with SWNT network after PAA electro-polymerization. **b**, Scanning electron microscopy (SEM) image showing some SWNTs not connected to the network, without polymer coating (after 50 CV cycles). **c-f**, SEM, images illustrating the effect of number of cyclic voltammetry (CV) electro-polymerization cycles (10, 30, 40, and 60) on polymer coating (1 mM AA used in EP process).

PAA thickness plays an important part in the resulting device sensitivity. Figure S1 illustrates that the optimal range of EP cycles creates a PAA coating that provides good coverage of the ox-SWNT but at the same time retains the individual SWNT structure. 0-10 EP cycles results in a PAA coating that is too thin while >60 EP cycles creates a coating so thick that the SWNT structure can no longer be visually detected in the SEM images.

Table S1: EDX data of an ox-SWNT/PAA device on a SiO_2 wafer (atomic %).

Carbon	Nitrogen	Oxygen	Silicon
34.48±1.57	7.02±3.77	18.68±11.89	39.82±14.56

EDX data shows the presence of four elements, however, with a large margin of error. Therefore, XPS experiments focused on carbon and nitrogen were performed to get an accurate ratio.

Table S2: **XPS data of an ox-SWNT device and a device functionalized with 41 EP cycles.**

Device	% Carbon	% Oxygen	% Nitrogen
ox-SWNTs	46.96±4.85	53.04±4.85	0
ox-SWNT/PAA	54.21±6.41	42.61±6.76	3.18±0.35

Table S2 shows that no nitrogen is present in the ox-SWNTs prior to EP functionalization. The high oxygen content arises from the oxidation state of the SWNTs and also from the microchip surface which consists of SiO_2. Pure poly(1-aminoantracene) has a carbon:nitrogen ratio of 14:1, equal to 7.14% nitrogen. The XPS data indicates one type of nitrogen present in the material with a binding energy of 398.7 eV (Fig S2). This binding energy corresponds to pyridinic nitrogen which analogues to nitrogen in PAA. The XPS data reveal a carbon:nitrogen ratio of 17:1 (5.55% nitrogen), this means there is approximately one monomer unit per three ox-SWNT carbons. Simple stoichiometry calculations agree with a weight ratio of approximately 5.4 mg PAA/mg ox-SWNTs.

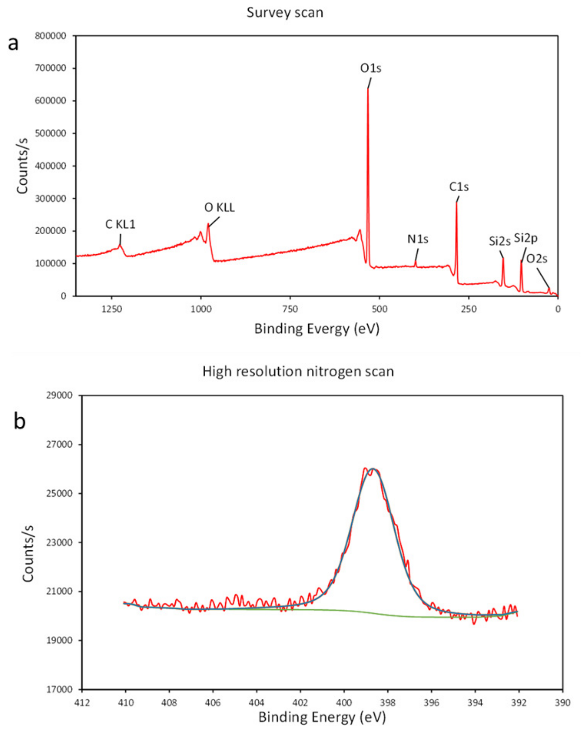

Figure S2: a, XPS survey scan of ox-SWNT/PAA device on microchip. **b**, High resolution N1s of the same device.

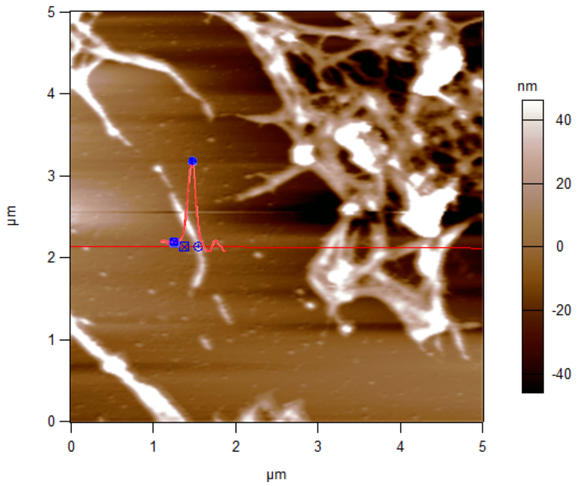

Figure S3: AFM image of ox-SWNT/PAA network. Overlay shows height profile of an individual ox-SWNT/PAA tube with a height of 35.65 nm.

Typical ox-SWNTs range from 2-5 nm in diameter, giving a polymer thickness of approximately 30 nm. Larger bundles of ox-SWNTs would form thicker polymer coatings.

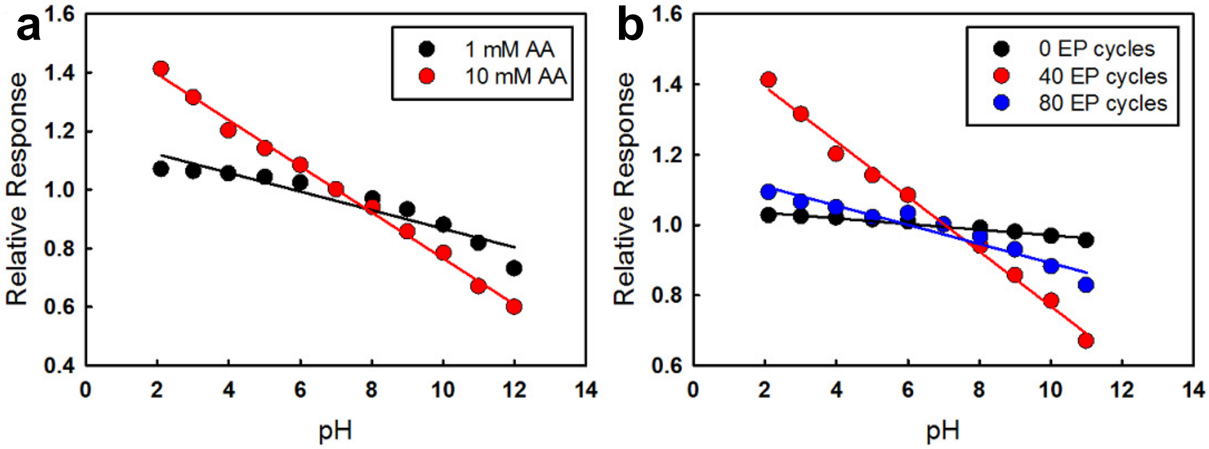

FigureS4. **a**, Calibration comparison of 40 EP cycles using 1 mM AA versus 10 mM AA. **b**, Calibration comparison of 0, 40, and 80 cycles using 10 mM AA.

The realization of the thickness dependent pH response for these ox-SWNT/PAA devices can also be used to optimize the concentration of monomer used during polymerization. pH responsive devices have been created using PAA in the past and it has been shown that the linearity of the response can be increased by using a 10 mM AA solution versus a 1 mM AA solution.[S1] Figure S4a compares a pH calibration, at 40 EP cycles, using these different AA concentrations. Based upon the r^2 value of the linear regression for 1 mM AA and 10 mM AA (0.886 and 0.995 respectively), the 10 mM AA concentration produces a more linear response over the 2 – 12 pH range. The determination of the optimal number of EP cycles holds true for this higher AA concentration (Figure S4b). For 0, 40, and 80 EP cycles the sentivities were 0.008 ± 0.001, 0.078 ± 0.002, and 0.027 ± 0.003 respectively.

S1 Faria, R. C. & Bulhões, L. O. S. Hydrogen ion selective electrode based on poly(1-aminoanthracene) film. *Anal. Chim. Acta* **377**, 21-27 (1998).

Table S3. The effect of acid treatment duration on SWNT properties and FET characteristics.

Sample	Length Distribution[a] (nm)	-COOH Loading[b] (μmol/mg)	Conductance at zero gate voltage (S)	ON/OFF ratio
Pristine	1898 ± 706	N/A	4.95 E-05	5.25
1 h oxidation	>1000	0.814 ± 0.128	1.52 E-03	3.46
1.5 h oxidation	750 ± 250	1.68 ± 0. 17	7.23 E-04	6.45
2 h oxidation	401 ± 137	2.88 ± 0.21	2.30 E-04	27.10
2.5 h oxidation	354 ± 150	3.46 ± 0.20	No conductance	---

a) Length distribution determined by TEM, b) carboxylic acid loading determined by Boehm's titration.

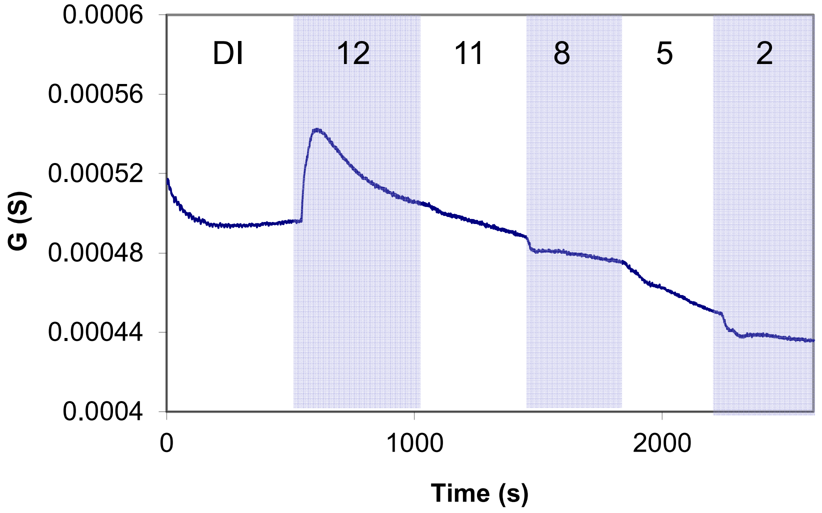

Figure S5. Change in conductance vs time of a device fabricated using pristine SWNTs functionalized with PAA (30 EP cycles) at various pH levels.

The signal response from pristine SWNT/PAA devices is adverse, and less than that of the ox-SWNT. This shows that hydrogen bonding between the carboxylic acid groups of the ox-SWNTs and the amine groups of the PAA provides signal enhancement of the device.

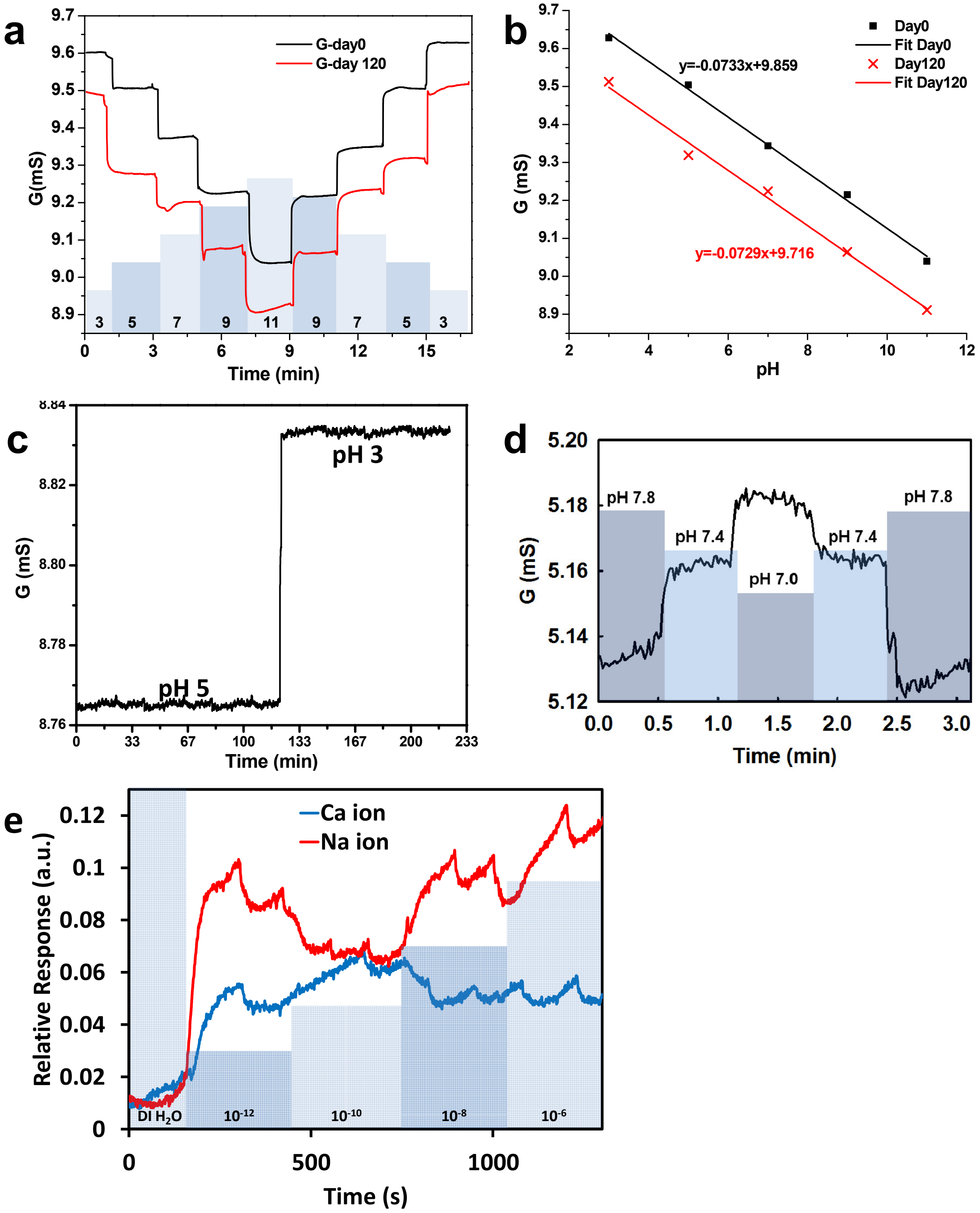

Figure S6. **Reproducibility and Stability of the pH Sensor. a**, Conductance vs. time measurements and **b**, the corresponding calibration curve (G vs. pH) of the same ox-SWNT/PAA device tested before (black) and after (red) it was shelf-stored for 120 days. **c**, Stability test of an ox-SWNTs/PAA device held in pH 5 buffer for 2 h then switched to pH 3 buffer for another 1.75 h. **d**, Sensitivity of ox-SWNT/PAA device in a physiologically relevant pH range. **e**, Cross-sensitivity of the device to Ca^{2+} and Na^{+} cations.

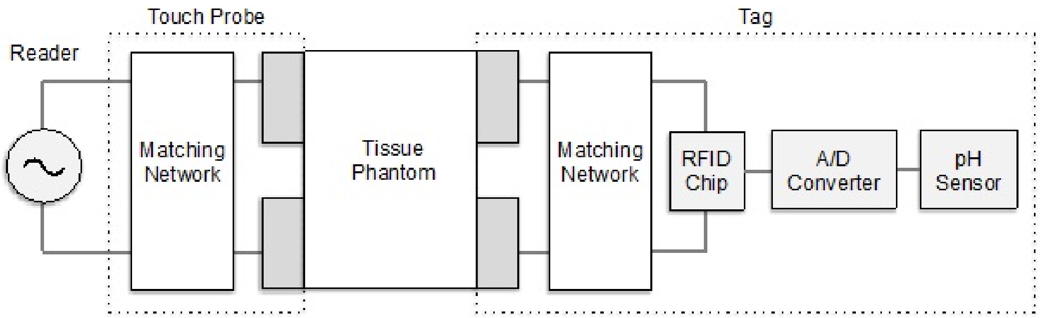

Figure S7. Block diagram of the wireless test setup

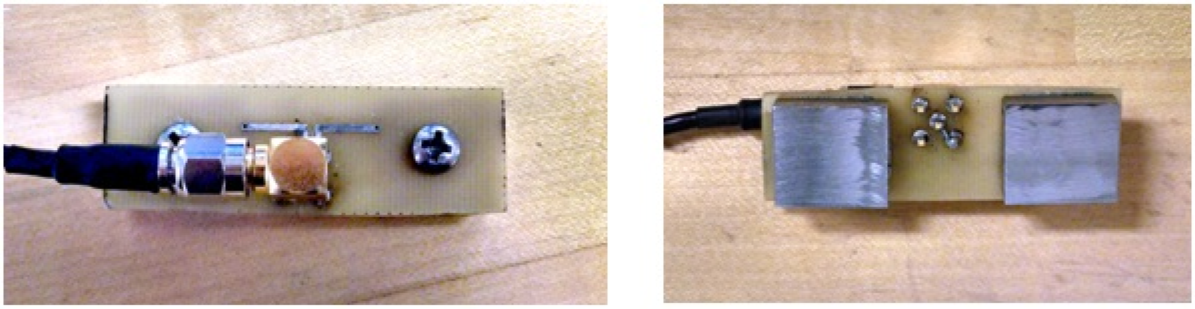

Figure S8. Custom touch probe top view (left), bottom view (right)

The implantable, RFID sensor system was tested according to the block diagram in Figure S7. The custom designed touch probe, shown in Figure S8, is comprised of an impedance matching network and two stainless steel electrodes. The purpose of the touch probe is to match the impedance of the RFID reader to that of the patient's tissue. In the test setup the electrodes are placed in contact with the tissue phantom directly over the implantable tag in order to wirelessly transmit power to, and communicate with the tag.

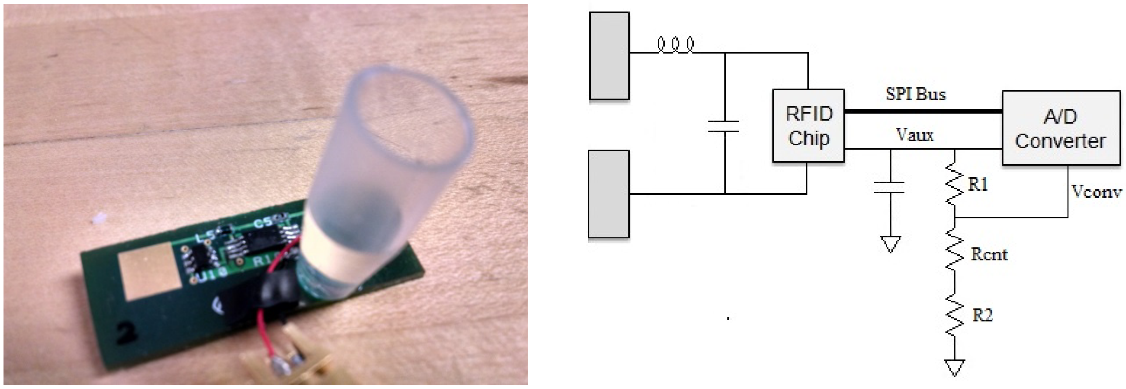

Figure S9. Fabricated implantable tag (left) and tag circuit diagram (right)

The implantable tag, shown in Figure S9, is a custom printed circuit board (PCB) that connects a commercially available RFID chip to an analog to digital converter (ADC) and a voltage divider network. Circuit board dimensions: 1.34 cm x 3.76 cm x 0.30 cm (with all electrical components installed. Since this is a passive tag, the RFID chip harvests power from the incoming RF energy and outputs a DC power source, Vaux, in order to power both the ADC and the voltage divider network. Rcnt in the circuit diagram below represents the carbon nanotube pH sensor. The remaining resistors, R1 and R2, comprise the rest of the voltage divider network. The relative level of the voltage across the sensor, Vconv, is related to the pH of the solution that surrounds the sensor. Vconv is then converted by the ADC to a digital representation and can be transmitted back to the RFID chip via a serial peripheral interface (SPI) bus.

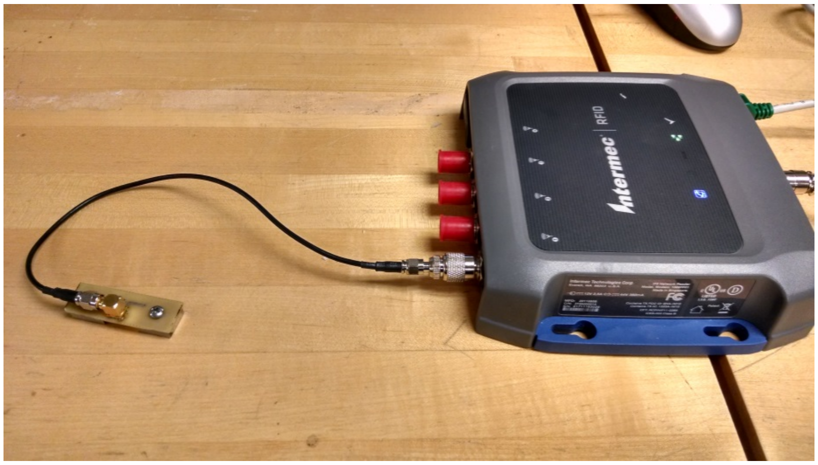

Figure S10. Custom touch probe connected to commercially available RFID reader

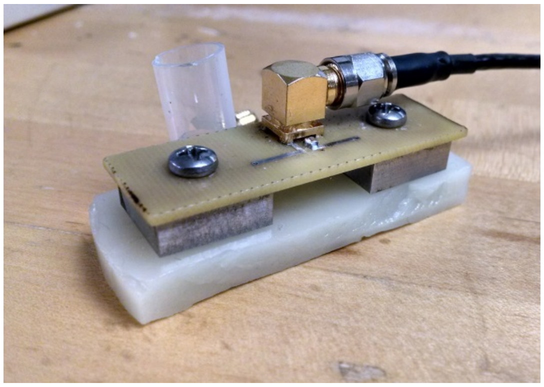

Figure S11. Touch probe in contact with the tissue phantom directly over the implantable tag

In the experimental setup the touch probe was connected to an Intermec IF2 Network Reader (Figure S10), and used to power and communicate with the tag through a 1-cm tissue phantom[S2] with properties similar to human skin (Figure S11). The sensor was exposed to buffer solutions of pH 5, pH 7.5, and pH 8.8 by placing the appropriate buffer solution in a plastic well attached to the tag.

S2 Porter, E. *et al.* in *Antennas and Propagation (EuCAP)*, 2010 Proceedings of the Fourth European Conference on. 1-5.

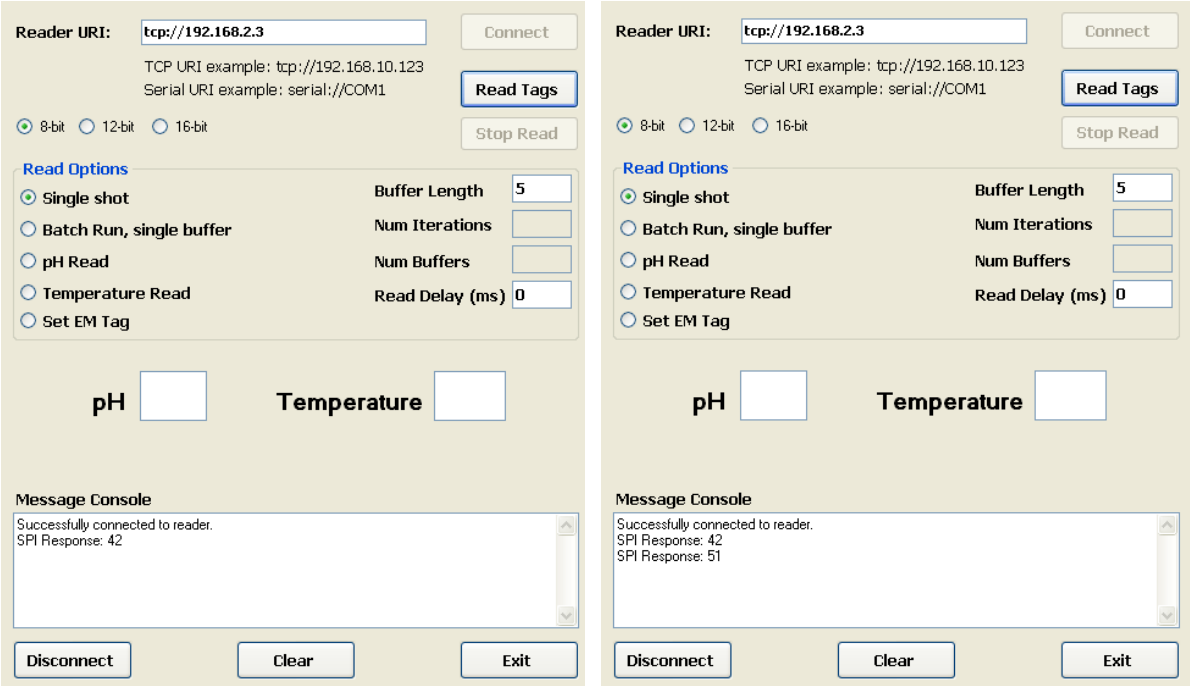

Figure S12. Custom GUI showing readings from the tag in response to solutions of pH 5 (left) and pH 8.8 (right)

A custom graphic user interface (GUI) was designed to display numerical values that were wirelessly read from the RFID chip on the tag. The values are inversely proportional to the sensor conductance. As is shown in Figure S12, the read value was 42 when the sensor was exposed to pH 5 (left), and increased to 51 when the sensor was exposed to pH 8.8 (right), indicating a detectable decrease in sensor conductance. The software can be calibrated to associate the numerical value with a pH value.

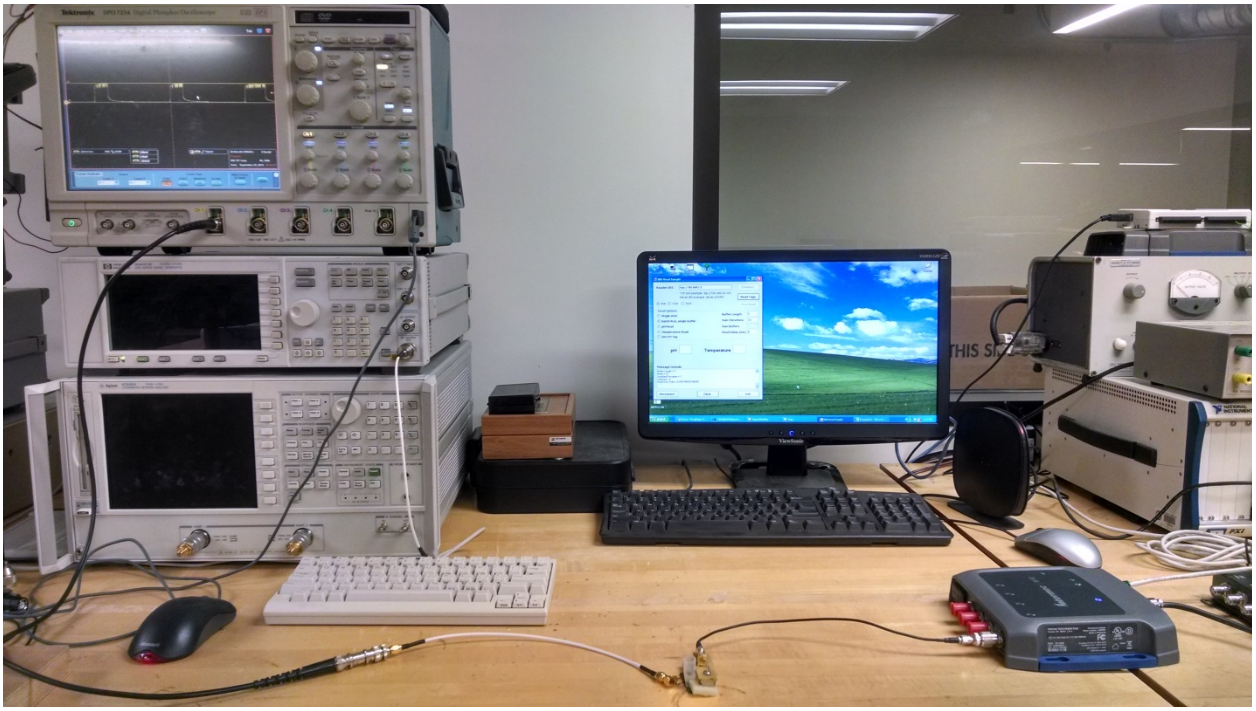

Figure S13. Complete testing setup

The voltage across the sensor was measured and also displayed on an oscilloscope while the tag was wirelessly powered using the touch probe. Figure S13 shows the complete testing setup.
